# Factors associated with death among hospitalized COVID-19 patients in Lagos State, Nigeria: a retrospective cross-sectional study

**DOI:** 10.4314/ahs.v22i3.52

**Published:** 2022-09

**Authors:** Olusola Adedeji Adejumo, Tope Ogunniyan, Adeife Valentina Adetola, Sandra Chizoba Mba, Olakunle Ogunbayo, Oluwaseun David Oladokun, Oluwayemisi Bamidele Oluwadun, Olufemi Erinoso, Sunday Adesola, Abimbola Bowale

**Affiliations:** 1 Mainland Hospital Yaba Lagos Nigeria; 2 Nigeria Centre for Disease Control. Nigeria Field Epidemiology and Laboratory Training Program Abuja Nigeria; 3 Department of Anaesthesia, Lagos State University College of Medicine Ikeja, Lagos Nigeria; 4 Department of Dentistry, Lagos State University Teaching Hospital Ikeja Lagos Nigeria

**Keywords:** COVID-19, Associated factors, deaths, Lagos, Nigeria

## Abstract

**Background:**

Lagos State has the highest burden of COVID-19 in Nigeria. We assessed associated factors with death from COVID-19 among hospitalized patients in Lagos, Nigeria.

**Methods:**

A retrospective cross-sectional study was conducted using de-identified records of laboratory-confirmed COVID-19 patients admitted into 15 isolation centers in Lagos State between February 27, 2020, and September 30, 2020.

**Results:**

A total of 2,858 COVID -19 patients were included in this study. The mean age of the patients was 41.9±15.5 years. A higher proportion of patients were males (65.8%), asymptomatic (55.5%), had no comorbid condition (72.2%) and had the mild disease (73.8%). The case fatality rate was 6.5%. The odds of death from COVID-19 infection increased by 4% with every increase in age (AOR 1.04, 95%CI 1.03–1.05, p<0.001). The chance of dying was 50% fold more among males (AOR 1.5, 95%CI 1.0 – 2.2, p = 0.042), 60% fold more among patients with comorbidity (AOR 1.6, 95%CI 1.3 – 2.4, p = 0.037) and 9 fold more among patients with severe COVID-19 infection (AOR 9.6, 95% CI 4.9 – 19.1, p <0.001).

**Conclusion:**

The odds of dying was higher among males, the elderly, patients with comorbidity and severe COVID-19.

## Introduction

Corona virus Disease 2019 (COVID-19) gained global attention when the World Health Organization (WHO) declared it a pandemic on March 11 2020. As of December 2020, there were over 62 million cases and 1.4 million deaths reported globally.^1^ In Africa, the total number of COVID-19 cases reported was over 2 million, with about 52,000 deaths as of December 2020, while Nigeria recorded 67,557 confirmed cases and 1,173 deaths.^2^ Lagos state, is the epicenter of the disease in Nigeria has the highest burden of COVID-19 confirmed cases in the country.^3^

Various factors have been identified as influencing poor outcomes among COVID-19 patients; these include but are not limited to being male, older age group, comorbidities, obesity, severe symptoms at presentation, and social deprivation.^4^–^6^ A systematic review involving 114 studies reported that older age, hypertension and diabetes increased the mortality risk of COVID-19 patients.^7^ Another systemic review and meta-analysis opined that mortality from COVID-19 was predicated on the mean age of patients.^8^

In Nigeria, a study conducted among 2184 patients with COVID-19 showed that the case fatality rate was 4.3%, the severity of symptoms and clinical signs at presentation were associated with mortality.^9^ Another Nigerian study among COVID-19 patients with comorbidity showed that the risk of death was highest among patients with hypertension and diabetes.^10^ Unlike previous studies from Lagos, Nigeria, the present study assessed the factors associated with death from COVID-19 in a larger and more diverse cohort of hospitalized patients, allowing for more generalizability of the study findings.

## Study Methods

### Study design

A retrospective cross-sectional study was conducted using de-identified medical records of laboratory-confirmed COVID-19 patients admitted into 15 isolation centers in Lagos State between February 27, 2020, and September 30, 2020.

### Study sites

The study was conducted in four private and eleven public treatment centers in Lagos established to handle inpatient care for COVID-19 patients in Lagos. Mainland hospital was the first treatment centre to manage COVID-19 patients being the only infectious disease hospital in Lagos state.^11^ Other centers were established as the number of COVID-19 cases increased. The private providers were involved in managing COVID-19 as alternatives for patients who prefer to be managed by private medical practitioners. All COVID-19 isolation centers had a standard treatment protocol developed by the Lagos State Government Incident Command System and the National Center for Disease Control (NCDC).^12^

### Study population

Based on the World Health Organization guidelines, all COVID-19 patients diagnosed using the real-time reverse transcription-polymerase-chain-reaction (RT-PCR) assay and admitted for treatment in Lagos State isolation centers were included in this study.^13^ Excluded from the study were suspected COVID-19 patients initially admitted into the isolation centers but later found to be negative for COVID-19 after RT-PCR assay, patients with inconclusive and missing results. COVID-19 positive patients on home-based care were also excluded from the study.

### Assessment of disease severity

Assessment of the severity of the disease was based on the NCDC guidelines.^12^ Mild cases are either asymptomatic or present with non-specific symptoms such as fever, cough, sore throat, nasal congestion, malaise, headache, muscle pain, loss of smell, loss of taste, diarrhea, vomiting and abdominal pain; with no evidence of viral pneumonia or hypoxia.^12^ Severe presentation in adults is characterized by high-grade fever (>380C) or suspected respiratory infection and one of the following: respiratory rate >30 breath/minute, severe respiratory distress, Spo2 < 90% in room air and any risk factor for severe infection. The risk factors for severe infection include being elderly (≥ 60 years), diabetes, hypertension, cardiac diseases, chronic lung disease, cerebrovascular disease, chronic kidney disease, immunosuppression, cancer and smoking.^14^

Severe infection in children is characterized by cough or difficulty in breathing and at least one of the following: central cyanosis or spo2 <92%, grunting respiration or severe respiratory distress, very severe in-drawing or signs of pneumonia.

### Testing practices

In Lagos, Nigeria, testing for COVID-19 was based on a strong suspicion of COVID-19 infection. Testing was done routinely for suspected cases of COVID-19. According to the NCDC, a suspect was any person with acute respiratory illness or new respiratory symptoms in the absence of an alternative diagnosis that explains the clinical presentation and a history of travel to or residence in a country reporting COVID-19 within 14 days prior to symptom onset. A COVID-19 suspect is any person with new respiratory symptoms who had contact with a confirmed or probable COVID-19 case in the last 14 days prior to symptom onset.^12^

### Admission and discharge criteria

The admission and discharge criteria changed over time with protocol reviews. At the initial stages, all diagnosed COVID-19 patients were admitted and discharged based on 2 consecutive negative samples and one negative sample later. After that, patients with mild symptoms were managed at home, provided the home environment was conducive for home treatment while the severe cases were managed in the hospital. Patients under home-based care were discharged after 14 days of isolation and treatment. All patients discharged after hospital care were asked to do self-isolation at home for one week.

Hospitalized patients were managed with Lopinavir-Ritonavir, Vitamin C, Vitamin D Zinc, blood thinners such as clopidogrel, Aspirin or Clexane, dexamethasone and intranasal oxygen depending on the severity. Patients were admitted into the intensive care unit when the Spo2 <90%. In addition, the patient's comorbid conditions were also managed along with the treatment for COVID-19.

The outcome variables were discharge or death. “Discharge" was based on the resolution of symptoms and a negative PCR-based SARS-CoV-2 virus test. Patients that died from the complications of COVID-19 while on admission were classified as “dead”. Discharged patients were seen at the clinic monthly for at least three months to ascertain their health status before discharge from follow-up.

### Data analysis

Patients' age, gender, presence and types of comorbidity, symptoms and risk factors, and outcome (Discharged or dead) were extracted from the central electronic medical records of all the isolation centers in Lagos State. Data was transported to IBM statistics for analysis. The severity of patients' presentation was determined using the NCDC guidelines. Continuous variables were presented as means and standard deviation, while percentages and frequencies were used to present categorical variables. The student ‘t’ test was used to compare the numerical variables of two independent groups, while the chi-squared test was used to compare proportions. Logistic regression was used to assess the factors associated with mortality among COVID-19 patients. The effect of possible confounders (symptoms and severity of disease) was reduced using regression analysis. Missing and incomplete data were excluded before data analysis. The confidence interval was set at 95% and p <0.05 was considered significant. IBM statistics version 26 was used for data analysis.

### Ethical Approval

The ethical approval was obtained from the Health Research Ethics Committee of the Lagos State University Teaching Hospital.

## Results

**Figure 1:**
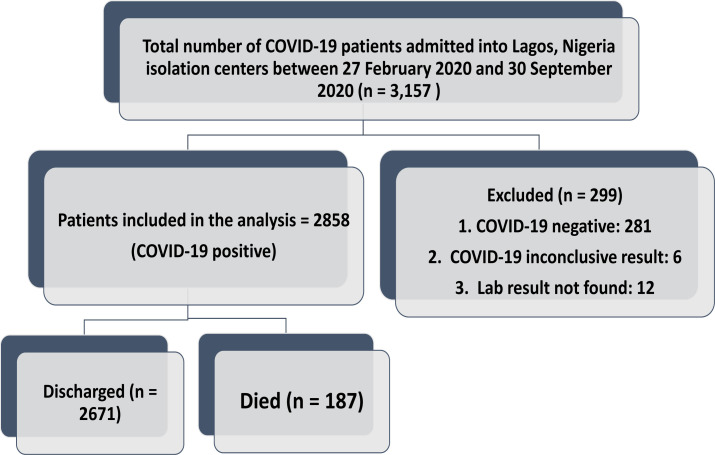
Patients' flow chart

A total of 3157 patients were admitted into Lagos isolation centers within the study period, out of which 299 (9.5%) were excluded. Of those excluded from the analysis, 281 (94.0%) were COVID-19 negative, 6 (2.0%) had an inconclusive result, and 12(4.0%) had no laboratory result (Figure 1). A total of 2,858 COVID -19 patients were included in this study. The mean age of the patients was 41.9 ±15.5 years, 42.0 % were aged between 20 - 39 years, while the proportion of patients aged below ten years and above 60 years was 2.0% and 13.4%, respectively. A higher proportion were male (65.8%), asymptomatic (55.5%), had no comorbid condition (72.2%) and had the mild disease (73.8%). The proportion of patients that died among those admitted was 6.5% (Table 1). The factors associated with mortality from COVID-19 are highlighted in Table 2. Mortality rates were highest among patients aged > 60 and 50–59 years (26.2% and 9.8% respectively), while there were no deaths in those less than ten years. The mean age of patients that died was higher (59.5± 14.5 years) than patients that survived (40.7±14, 8 years) (p<0.001). The mortality rate was higher among the males (7.3% vs. 5.1%, p= 0.026), symptomatic patients (13.0% vs. 1.3%, p <0.001), patients with comorbidity (16.5% vs. 2.7%, p<0.001) and patients with severe presentation (22.3% vs 0.9%, p<0.001).

**Table 1:** Socio-demographic and clinical details of COVID-19 patients

Variable	Frequency (n = 2858)	%
Age group ( in years)		
< 10	57	2.0
10 – 19	84	2.9
20 – 29	443	15.5
30 – 39	756	26.5
40 – 49	687	24.0
50 – 59	449	15.7
≥ 60	382	13.4
**Mean±SD**	41.8±15.5	

Gender		
Male	1,880	65.8
Female	978	34.2

Comorbidity		
Yes	795	27.8
No	2,063	72.2

Severity		
Mild	2,106	73.8
Severe	752	26.2

Presence of symptoms		
Yes	1,273	44.5
No	1,585	55.5

Presence of risk factors		
Yes	909	31.8
No	1,949	68.2

Outcome		
Discharged home	2,671	93.5
Died	187	6.5

SD: standard deviation.

**Table 2:** Factors associated with mortality from COVID-19

Variable	Final Outcome		
	Discharge	Died	p-value
	n = 2,671 (%)	n = 187 (%)	
Age group (in years)			
< 10	57 (100.0)	0 (0.0)	
10 – 19	83 (98.8)	1 (1.2	
20 – 29	437 (98.6)	6 (1.4)	
30 – 39	746 (98.7)	10 (1.3)	
40 – 49	661 (96.2)	26 (3.8)	
50 – 59	405 (90.2)	44 (9.8)	
≥ 60	282 (73.8)	100 (26.2)	
Mean±SD	40.7±14.8	59.5± 14.5	<0.001

Gender			
Male	1,743 (92.7)	137 (7.3)	
Female	928 (94.9)	50 (5.1)	0.026

Presence of symptoms			
Yes	1,107 (87.0)	166 (13.0)	<0.001
No	1,564 (98.7)	21 (1.3)	

Number of symptoms			
None	1,564 (98.7)	21 (1.3)	<0.001
1	488 (87.6)	69 (12.4)	
2	409 (89.1)	50 (10.9)	
≥ 3	210 (81.7)	47 (18.3)	

Has comorbidity			
Yes	664 (83.5)	131 (16.5)	<0.001
No	2,007 (97.3)	56 (2.7)	

Number of comorbidities			
None	2,007 (97.3)	56 (2.7)	<0.001
1	497 (87.2)	73 (12.8)	
2	153 (74.6)	52 (25.4)	
≥ 3	14 (70.0)	6 (30.0)	

Disease severity			
Mild	2,087 (00.1)	19 (0.9)	<0.001
Severe	584 (77.7)	168 (22.3)	

SD: standard deviation.

The odds of death from COVID-19 infection increased by 4% with every increase in age (AOR 1.04, 95% CI 1.03 – 1.05, p<0.001). Compared with the females, the odds of dying was 50% fold more in males (AOR 1.5, 95% CI 1.0 – 2.2, p = 0.042). In addition, the odds of mortality was 60% fold more among patients with comorbidity (AOR 1.6, 95% CI 1.3 – 2.4, p = 0.037) than patients without comorbidity, while it was over nine-fold more among patients with severe COVID-19 infection than patients with mild presentation (AOR 9.6, 95% CI 4.9 – 19.1, p <0.001) (Table 3)

**Table 3:** Regression analysis of factors associated with COVID-19 mortality

Variable	AOR	95% CI	p-value
Age (in years)	1.04	1.027 – 1.053	<0.001

Female	1		
Male	1.5	1.0 – 2.2	0.042

Asymptomatic	1		
Symptomatic	2.4	0.9 – 5.7	0.071

No comorbidity	1		
Comorbidity	1.5	1.0 – 2.2	0.037

Mild	1		
Severe	9.6	4.9 – 19.1	<0.001

CI: confidence interval, AOR: adjusted odds ratio

Of the risk factors for severe COVID-19, Age (AOR 1.1, 95% CI 1.0–1.1, p<0.001), Diabetes (AOR 2.2, 95% CI 1.5 – 3.3, p<0.001), HIV (AOR 7.4, 95% CI 2.7 - 20.3, p<0.001) and Cancer (AOR 6.4, 95% CI 1.8 – 22.9, p=0.004) were associated with mortality. (Table 4)

**Table 4:** Risk factors as predictors of COVID-19 mortality

Variable	AOR	95% CI	p-value
Age (in years)	1.1	1.0 – 1.1	<0.001
Hypertension	1.4	1.0 – 2.1	0.075
Diabetes	2.2	1.5 – 3.3	<0.001
Chronic lung disease	1.8	0.9 – 3.9	0.116
Chronic kidney disease	1.9	0.5 – 6.4	0.324
Immunosuppression	7.4	2.7 – 20.3	<0.001
Cancer	6.4	1.8 – 22.9	0.004

CI: confidence interval, AOR: adjusted odds ratio

## Discussion

Since the first case of COVID-19 was reported in Nigeria on February 27 2020, Lagos State has actively managed many PCR confirmed COVID-19 cases at its various isolation and treatment centers. In this study, most hospitalized patients were adults aged 20 years and above, with about 5% aged less than 20 years. This finding aligns with other studies^15^,^16^ and suggests that older adults are more susceptible to COVID-19. This study also demonstrated that males were predominantly affected, and the majority of the cases were asymptomatic or had mild symptoms. Similar findings have been reported in other studies among hospitalized COVID-19 patients.^17^–^19^ In the cases reviewed, comorbidities such as hypertension and diabetes were relatively common. This finding corroborates the current evidence that comorbidities are common in COVID-19 patients and are a reason for hospitalization and increased disease severity.^20^–^22^

In this study, the mortality rate in the hospitalized COVID-19 cases was 6.6%. While varying rates have been documented in different regions of the world, the relatively low proportion, as seen in this study, conforms to the low mortality rates reported on the African continent.^2^,^23^ In older patients, COVID-19 has been characterized by disease severity, poor outcome, and higher mortality.^15^,^19^,^21^ Our study likewise supported this fact as most deaths were recorded in the older age groups, especially those aged 60 years and above. Being male has been identified as a factor associated with poor outcomes and mortality from COVID-19.^19^,^22^–^25^ In this study, similar results were observed as male patients had higher mortality rates compared to females. This finding can be explained by a decrease in the efficiency of immune response with increasing age and the ability for females to mount more robust adaptive and innate immune responses than males.^26^ The presence of comorbidities such as cardiovascular diseases, diabetes mellitus, and obesity has been documented widely to be associated with severe illness, poor outcome and mortality among COVID-19 patients.^19^, ^27^–^29^ As in previous studies,^19^, ^27^–^29^ findings from this study showed that the mortality rate was higher among patients with a prior history of hypertension, diabetes, cancer, chronic kidney disease and chronic lung diseases, with the rates increasing in patients with multiple comorbidities. Patients with severe COVID-19 disease have been reported to exhibit acute respiratory distress syndrome (ARDS), acute respiratory failure, acute renal injury, multi-organ failure, and may eventually die.^21^,^30^,^31^ An essential factor associated with the severity and prognosis of COVID-19 disease is the increased release of systemic pro-inflammatory cytokines and other inflammatory markers indicative of a phenomenon called “cytokine storm”. This phenomenon can be exacerbated by underlying systemic illnesses and may explain the poorer prognosis in cases with comorbidities.^32^, ^33^ Similar findings were observed in this study as patients with comorbidities had higher mortality rates.

## Limitations

Our study had some limitations. Firstly, the meticulous and thorough collection of information was complex during the first pandemic wave, given the rapid surge of cases, the fear of contracting the disease and the little knowledge about the disease. Second, data on underlying health conditions and comorbidities that could increase the risk for complications and severe illness were self-reported and inaccessible from some critically ill patients. Limited testing and admission process in the State may have resulted in the under-reporting of cases and selection bias. Furthermore, we had no access to the variant typing done in the State. Thus, we could not determine if there was an association between the variant and the severity of the illness.

Nonetheless, the current study provides additional evidence and validates prior findings of risk factors associated with mortality in COVID-19 disease. The

## Conclusion

This study explored the factors associated with poor outcomes among COVID-19 patients in the isolation centers in Lagos State. With poorer outcomes and higher mortality rates among men, older persons, those with comorbidities, and severe COVID-19, public health measures to prevent the transmission of the SARS-CoV-2 virus should be sustained, particularly among these groups. Social distancing, use of facemasks, and regular hand-washing are recommended to slow the spread of the virus and help protect vulnerable older adults. Early vaccination will reduce the risk of severe infection and mortality,

## References

[1] World Health Organization (2020). Naming the coronavirus disease (COVID-19) and the virus that causes it.

[2] African Union and African CDC (2020). Coronavirus Disease 2019 (COVID-19). Latest Updates on the COVID-19 Crisis from Africa CDC.

[3] Nigeria Center for Disease Control COVID-19 tracking dashboard for Nigeria.

[4] Velavan TP, Meyer CG (2020). The COVID-19 epidemic. Trop Med Int Health.

[5] Martins-Filho PR, Tavares CSS, Santos VS (2020). Factors associated with mortality in patients with COVID-19. A quantitative evidence synthesis of clinical and laboratory data. Eur J Intern Med.

[6] Grasselli G (2020). Risk factors associated with mortality among patients with COVID-19 in intensive care units in Lombardy, Italy. JAMA Intern Med.

[7] Mehraeena E, Karimib A, Barzegaryc A, Vahedib F, Afsahid AM, Dadrase O (2020). Predictors of mortality in patients with COVID-19-a systematic review. Eur J Integr Med.

[8] Mesas AE, Cavero-Redondo I, A’ lvarez-, Bueno C, Sarria’ Cabrera MA, Maffei de Andrade S (2020). Predictors of in-hospital COVID-19 mortality: A comprehensive systematic review and meta-analysis exploring differences by age, sex and health conditions. PLOS ONE.

[9] Abayomi A, Odukoya O, Osibogun A, Wright O, Adebayo B, Balogun M (2021). Presenting symptoms and predictors of poor outcomes among 2,184 patients with COVID-19 in Lagos State, Nigeria. Int J Infect Dis.

[10] Osibogun A, Balogun M, Abayomi A, Idris J, Kuyinu Y, Odukoya O (2021). Outcomes of COVID-19 patients with comorbidities in southwest Nigeria. PLoS ONE.

[11] Bowale A, Abayomi A, Idris J, Omilabu S, Abdus-Salam I, Adebayo B (2020). Clinical presentation, case management and outcomes for the first 32 COVID-19 patients in Nigeria. Pan Afr Med J.

[12] Nigeria Centre for Disease Control National Interim Guidelines for Clinical Management of COVID-19.

[13] (2020). Laboratory testing strategy recommendations for COVID-19.

[14] World Health Organisation COVID-19 Clinical management: living guidiance.

[15] Guan W, Ni Z-Y, Hu Y, Liang W, Ou C, He J (2020). Clinical characteristics of coronavirus disease 2019 in China. N Engl J Med.

[16] Davies NG, Klepac P, Liu Y, Prem K, Jit M, Eggo RM (2020). Age-dependent effects in the transmission and control of COVID-19 epidemics. Nat Med.

[17] Channappanavar R, Zhao J, Perlman S (2014). T cell-mediated immune response to respiratory coronaviruses. Immunol. Res.

[18] Nelemans T, Kikkert M (2019). Viral innate immune evasion and the pathogenesis of emerging RNA virus infections. Viruses.

[19] Zhou F, Yu T, Du R, Ren L, Zhao J, Hu Y (2020). Clinical course and risk factors for mortality of adult in patients with COVID-19 in Wuhan, China: a retrospective cohort study. Lancet.

[20] Jordan RE (2020). Covid-19: risk factors for severe disease and death. BMJ.

[21] Huang C, Wang Y, Li X (2020). Clinical features of patients infected with 2019 novel coronavirus in Wuhan, China. Lancet.

[22] Liang WH, Guan WJ, Li CC, Li YM, Liang HR, Zhao Y (2020). Clinical characteristics and outcomes of hospitalised patients with COVID-19 treated in Hubei (epicentre) and outside Hubei (non-epicentre): a nationwide analysis of China. Eur Respir J.

[23] World Health Organisation (2020). Coronavirus Disease (COVID-19) Situation Report -106.

[24] Richardson S, Hirsch JS, Narasimhan M, Crawford JM, McGinn T, Davidson KW (2020). Presenting characteristics, comorbidities, and outcomes among 5700 patients hospitalised with COVID-19 in the New York City area. JAMA.

[25] Jin JM, Bai P, He W, Wu F, Liu XF, Han DM (2020). Gender Differences in Patients With COVID-19: Focus on Severity and Mortality. Front Public Health.

[26] Rapp JL, Lieberman-Cribbin W, Tuminello S, Taioli E (2021). Male Sex, Severe Obesity, Older Age, and Chronic Kidney Disease Are Associated With COVID-19 Severity and Mortality in New York City. Chest.

[27] Verity R, Okell LC, Dorigatti I, Winskill P, Whittaker C, Imai N (2020). Estimates of the severity of coronavirus disease 2019: a model-based analysis. Lancet Infect Dis.

[28] Mehra MR, Desai SS, Kuy SR, Henry TD, Patel AN (2020). Cardiovascular disease, drug therapy, and mortality in Covid-19. N Engl J Med.

[29] Du RH, Liang LR, Yang CQ, Wang W, Cao TZ, Li M (2020). Predictors of mortality for patients with COVID-19 pneumonia caused by SARS-CoV-2: a prospective cohort study. Eur. Respir. J.

[30] Li L, Huang T, Wang Y (2020). 2019 novel coronavirus patients' clinical characteristics, discharge rate and fatality rate of meta-analysis. J Med Virol.

[31] Chen N, Zhou M, Dong X (2020). Epidemiological and clinical characteristics of 99 cases of 2019 novel coronavirus pneumonia in Wuhan, China: a descriptive study. Lancet.

[32] Chen G, Wu D, Guo W, Cao Y, Huang D, Wang H (2020). Clinical and immunological features of severe and moderate coronavirus disease 2019. J Clin Invest.

[33] Qin C, Zhou L, Hu Z, Zhang H, Yang S, Tao Y (2020). Dysregulation of Immune Response in Patients with Coronavirus 2019 (COVID-19) in Wuhan, China. Clin Infect Dis.

